# A conceptual model of barriers and facilitators to primary clinical teams requesting pediatric palliative care consultation based upon a narrative review

**DOI:** 10.1186/s12904-019-0504-8

**Published:** 2019-12-21

**Authors:** Jennifer K. Walter, Douglas L. Hill, Concetta DiDomenico, Shefali Parikh, Chris Feudtner

**Affiliations:** The Children’s Hospital of Philadelphia, 2716 South St 11th Floor, Philadelphia, PA 19146 USA

**Keywords:** Pediatric palliative care, Patient care team, Group processes, Communication inhibitors

## Abstract

**Background:**

Despite evidence that referral to pediatric palliative care reduces suffering and improves quality of life for patients and families, many clinicians delay referral until the end of life. The purpose of this article is to provide a conceptual model for why clinical teams delay discussing palliative care with parents.

**Discussion:**

Building on a prior model of parent regoaling and relevant research literature, we argue for a conceptual model of the challenges and facilitators a clinical team might face in shifting from a restorative-focused treatment plan to a plan that includes palliative aspects, resulting in a subspecialty palliative care referral.

Like patients and families, clinicians and clinical teams may recognize that a seriously ill patient would benefit from palliative care and shift from a restorative mindset to a palliative approach. We call this transition “clinician regoaling”. Clinicians may experience inhibitors and facilitators to this transition at both the individual and team level which influence the clinicians’ willingness to consult subspecialty palliative care. The 8 inhibitors to team level regoaling include: 1) team challenges due to hierarchy, 2) avoidance of criticizing colleagues, 3) structural communication challenges, 4) group norms in favor of restorative goals, 5) diffusion of responsibility, 6) inhibited expression of sorrow, 7) lack of social support, 8) reinforcement of labeling and conflict. The 6 facilitators of team regoaling include: 1) processes to build a shared mental model, 2) mutual trust to encourage dissent, 3) anticipating conflict and team problem solving, 4) processes for reevaluation of goals, 5) sharing serious news as a team, 6) team flexibility.

**Conclusions:**

Recognizing potential team level inhibitors to transitioning to palliative care can help clinicians develop strategies for making the transition more effectively when appropriate.

## Background

Referral to pediatric palliative care reduces suffering and improves the quality of life for these children while providing support to family members [1, 2]. Despite pediatric palliative care programs becoming more available [3, 4], many clinicians do not refer children to palliative or hospice care before they die [5] or refer them only late in the course of the disease, greatly limiting the potential benefits of palliative and hospice services [6].

In this paper we provide a set of reasons why teams may delay consultation, following along with a fictionalized illustrative case.

## Methods

We developed a conceptual model for the team level inhibitors and facilitators of regoaling based upon our clinical experience and social psychology theories of individual and group behavior [7–18]. We then conducted a narrative review of literature in the fields of palliative care, organizational psychology, and social psychology to identify relevant team behaviors around changes in team goals and team function. Searches were conducted in PubMed and PsycINFO for relevant articles.

## Discussion

### REGOALING

*Sara, a healthy toddler, suffered a near drowning accident at a neighbor’s pool two days ago. Due to the lack of oxygen, her brain was severely injured. She will never walk or talk again. Despite being overwhelmed by all of her new medical complexity, her parents are hopeful she will still have a good quality of life and want to continue life-sustaining treatments.*

For many pediatric conditions, parents and clinicians have a good reason to maintain hope for a cure or return to baseline function, and teams caring for these children describe the importance of supporting parents in hopeful thinking [19, 20]. Some patients, however, experience a gradual decline of function and quality of life. Our previous research suggests that parents who experience higher levels of negative affect and hopeful thinking (a general belief that one is usually capable of accomplishing goals and generating new goals if some goals are blocked), along with a necessary level of positive affect, are more likely over time to reevaluate their initial restorative seeking goals and replace them with new goals [21]. Based on these findings, we have suggested that parents of seriously ill children can undergo a process of regoaling: disengaging from a set of restorative goals that are no longer attainable or desirable and reengaging in a new set of more attainable goals such as keeping the child comfortable [22]. For regoaling to occur, the clinical team may need to suggest transitioning away from exclusively restorative-seeking treatments to focus on reducing the child’s suffering while maintaining quality of life. Referral by the primary clinical team to sub-specialty palliative care may facilitate this regoaling.

**Fig. 1:**
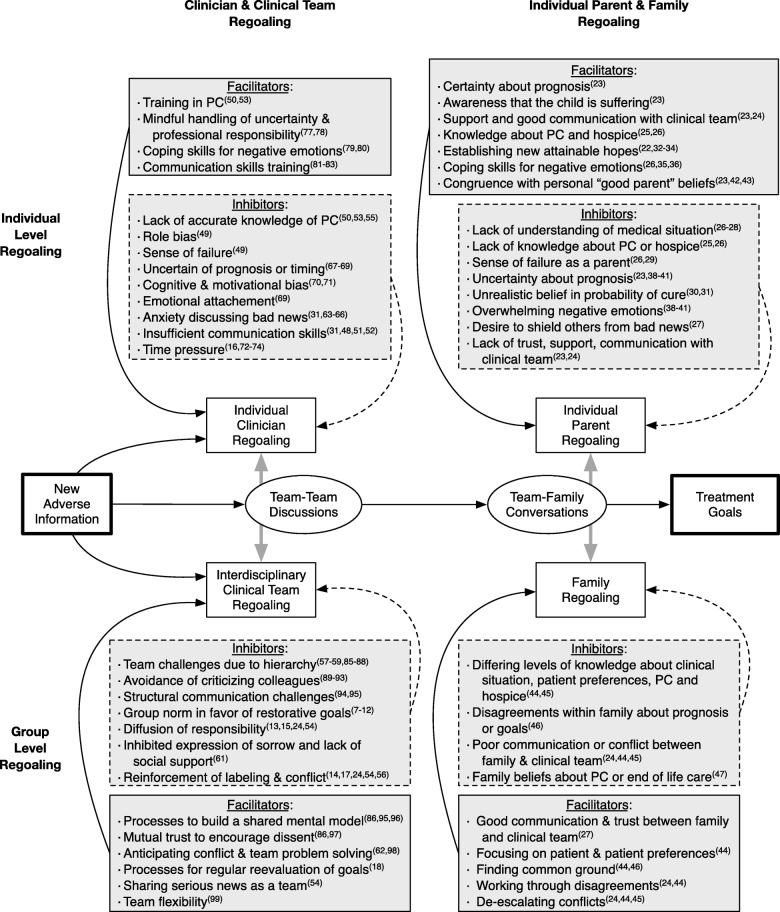
Conceptual model of facilitators and inhibitors to regoaling and consulting palliative care among individual clinicians, clinical teams, individual parents, and families

We suggest that teams of clinicians must go through a similar process of regoaling for them to consider subspecialty palliative care (Fig. 1 left bottom quadrant). Researchers have done much work to identify inhibitors to initiating palliative care at the individual parent level [21–43], at the family level [24, 27, 44–47] (Fig. 1 right half) and at the individual clinician level [31, 48–55] (Fig. 1 upper left quadrant), but less work has been done on factors that inhibit or facilitate at the team level [24, 56–59] and additional factors that promote or inhibit teams’ ability to communicate this recommendation to families [31, 51, 59–62]. Because most care for complex patients happens in interprofessional teams, and the team dynamic substantially influences decisions like consultation with subspecialty palliative care, we have primarily focused on team level factors affecting consultation rather than individual level factors. The focus of this paper is to explore reasons why teams of clinicians may neglect to either consider initiating palliative care or discussing palliative care with parents.

### Individual clinician inhibitors and facilitators to REGOALING

Individual clinicians may experience several inhibitors to considering palliative care (Fig. 1 upper left quadrant). Many pediatric clinicians lack accurate knowledge of palliative care services or believe that palliative care is primarily for families who have decided to move completely to comfort care [50, 53, 55]. Physicians may perceive that their role is to offer restorative treatment options [49]. Some physicians experience a sense of failure when they are unable to cure patients and report that discussing issues like palliative care and end of life issues with families is difficult [31, 49, 63–66]. When clinicians are uncertain of the patient’s prognosis, they may put off goals of care discussions for too long while waiting for more definitive information to provide to families [67–69]. Confirmation bias (a kind of cognitive bias) reduces the likelihood that doctors will question their initial diagnoses and treatment plan [70] and may make them less likely to discontinue a treatment once it has been initiated even if the treatment is no longer effective [71]. Another inhibitor to palliative care referrals may be the personal attachment between clinicians, patients, and family members [69]. Clinicians may worry that they are abandoning the patient if they refer them to palliative care. Many physicians report that breaking bad news and discussing issues like palliative care and end of life issues with families is difficult, stressful, and unsatisfying [31, 63–66]. This anxiety is worsened by the fact that clinicians acknowledge that they received limited or no communication training in how to share bad news or empathize with an upset parent [31, 48, 51, 52]. Clinicians are also under considerable time pressure to see a certain number of patients and families each day which may both increase the cognitive biases mentioned above [72–74], reduce their ability to consider alternative approaches [16], and make them reluctant to initiate difficult conversations about palliative care that may take an unknown amount of time [61, 75, 76]. Each of these factors may increase the likelihood of an individual clinician postponing discussing palliative care with families.

While these individual inhibitors exist, there are also individual level facilitators which support clinician regoaling (Fig. 1 upper left quadrant) like training in primary palliative care [50, 53], mindful handling of uncertainty [77, 78], practice of positive coping skills for negative emotions [79, 80], as well as communication skills training [81–83].

### Team level inhibitors to REGOALING

5 years later

*The pulmonology social worker, who had followed the family for years, suggested that the medical team consult the palliative care team to support the parents in the tracheostomy decision given her increased frequency and severity of central apneic episodes. The social worker had heard the parents say in previous admissions that they were unsure what the right thing to do was now that Sara kept getting hospitalized. The attending pulmonologist and intensivist were reluctant to consult palliative care. To them, the family had indicated they wanted “everything done” to help her over this acute episode and the attendings worried the family would think they were giving up on Sara if they suggested palliative care.*

Interprofessional teams are the standard of care for complex patients like children with cancer, or hypoxic brain injury, like Sara [58, 84]. High functioning teams can collaborate in care plan development and execution of patient care. Ideally, each team member contributes their expertise and the overall team engages in a collaborative, iterative decision-making process engaging healthcare professionals, patients, and families [84]. How teams navigate differences in opinion about what to offer families is an important indicator of team function. The following are potential inhibitors that teams may experience in coming to the decision to offer palliative care to a family.

#### Team communication challenges due to hierarchy

Team based care can lead to communication breakdowns between clinicians within a given team and between teams of different disciplines (e.g., intensive care and pulmonology) [57–59]. Attendings may fail to discuss with other team members such as nurses or occupational therapists important prognostic information or goals of care conversations that they have had with families [85]. Team members with an inaccurate mental model [86] of the patient’s condition are unable to contribute meaningfully to the care plan development, and may feel as though the team thinks their contribution isn’t essential. Rigid hierarchical structures may make it difficult for traditionally lower status team members (e.g. nurses and social workers) to share important information with higher status team members (e.g. physicians) [87]. Time pressure can enhance the tendency of a small number of group members to dominate the decision-making [88]. The traditional structure of many hospitals where attending physicians come onto a unit for limited periods of time may enhance communication problems if attending physicians do not seek out information from team members who have worked with a family for an extended period of time. In Sara’s case, team members perceived the social worker’s concerns as less relevant than the attending’s experience despite the fact that the social worker had the longest standing relationship with the family.

#### Avoidance of the perception of criticizing a colleague’s decision

Team members may also avoid challenging the clinical decision-making of their peers [89, 90]. Clinicians are wary of indicating that their colleagues have made a poor decision, perhaps because of concern that others will judge their decision-making in the future. Clinicians may also worry that their legitimate concerns will be misperceived as a personal attack causing colleagues to react badly [91, 92]. While clinicians report shared norms about not disagreeing with colleagues in front of a patient, many clinicians describe not knowing how to disagree appropriately behind closed doors [93].

#### Structural challenges to team communication

Structural factors may worsen teams’ abilities to share information and make decisions together. In a study in a neonatal intensive care unit, physicians expressed concern that rounds and meetings did not consistently include all team members [94, 95]. Rounds or interprofessional weekly team meetings may be limited to updates about each patient’s current diagnosis and treatment without much open discussion of alternative treatment paths.

Decisions about a potential transition to palliative care may also take place in informal settings outside of the team meetings. These conversations rarely occur with the whole team present either on an inpatient floor or in an outpatient clinic because the relevant clinicians have patient care responsibilities spanning large physical areas. In many instances, clinicians may only engage with a limited number of team members that they feel comfortable with. These teams may miss the perspectives and relevant information from other team members about what the family understands, what the family is worried about, and how the family might respond to the new information and a recommendation of palliative care.

#### Group norm in favor of restorative-seeking treatment

Group dynamics can enhance the individual biases in favor of restorative focused treatment. In ambiguous situations, groups can converge on persistent norms based on arbitrary suggestions [7, 8]. Group members may also fail to share new critical information and persist at ineffective strategies when a problem changes [9, 10]. Cohesive groups in stressful situations may engage in a variety of maladaptive processes also known as “groupthink” (e.g., self-censorship, illusion of unanimity, pressure on dissenters to conform) to reach a decision that ignores contrary evidence [11, 12]. In teams with little mutual trust, dissenters may be reluctant to challenge group norms toward aggressive treatment.

#### Diffusion of responsibility

Clinical teams may also experience diffusion of responsibility, when no one individual takes responsibility for doing something to change the situation [13, 15]. Clinicians may defer that responsibility to another provider who “knows the patient better.” Some clinicians may strongly believe that cure-seeking treatments are no longer helping the patient, but see it as outside their role to raise such issues [54]. Different team members may convey different direct or implied messages to the family increasing the confusion of the family [24]. Mixed messages and passive disapproval within the team about what is communicated to the family can set the stage for misunderstandings and conflicts between clinicians and families.

#### Inhibited expression of sorrow/lack of social support

Often the acknowledgement that a patient has a worsening clinical trajectory and may benefit from subspecialty palliative care will elicit feelings of sorrow among clinicians. However, many clinical teams have group norms about avoiding significant expressions of sorrow, and thus the topics which may elicit them, leading them to avoid discussion at the group level. Clinicians may maintain these norms because of their desire to retain composure professionally as well as their need during the clinical day to continue the fast-paced care of other patients with little to no time allotted to dwelling on these difficult situations [61]. Such norms can make it harder for individual team members to seek support when coping with negative emotions and may increase the risk for depression and burnout among clinicians. These norms of behavior are often ingrained early in training and individuals who are too emotional receive implicit and explicit messages that they need to learn to “cope” differently.

#### Labeling parents and escalating conflict

Some clinicians report that the main inhibitor to initiating difficult goals of care discussions is that the patients and family were not “ready.” [54] This perception may be the result of parents having responded in the past to difficult news with anger or an insistent request for more treatment. Clinicians may treat these reactions as fixed, unchangeable traits of the family, instead of recognizing that many families undergo a gradual transition from one set of goals to another [22]. When teams develop negative beliefs about a person, whether based on stereotypes or past experience, these beliefs may become self-fulfilling prophecies, especially if an individual is expected to be hostile [14, 17].

Clinical teams who see family members a problem to be overcome may end up rigidly adhering to their position and issuing ultimatums to the family [56]. Conflicts can escalate from mild cases of insensitive communication to severe cases where the conflict between clinicians and parents becomes the central focus instead of the child [24].

### Facilitators of team REGOALING

2 years further along

*Sara, having received her tracheostomy, has required more frequent hospitalizations. At her next hospitalization, the primary intensivist agreed to call a team meeting to discuss her gradual decline. The social worker had engaged her parents in conversations about preliminary goals of care and thought they would benefit from learning about out of hospital do-not-resuscitate orders and even possibly enrolling in hospice. Sara’s neurologist worried that her family would get the wrong impression if palliative care was mentioned. He worried that the team was giving them an unintended message that the medical team had given up. The palliative care team met with John and Maria and learned that they were increasingly worried about Sara’s suffering and feared she would die in the hospital when they weren’t there. Her family was not ready to sign a physician’s order for life sustaining treatment (POLST) form or enroll in hospice, but they made a plan to see the palliative care team at their next outpatient pulmonary visit. The parents said they appreciated the support from the palliative care team and they were relieved to finally be able to talk about some of their fears.*

There are a number of strategies that can help teams work together and to consider palliative care before patients are close to death. These include: a) building and maintaining a shared model of the situation, b) increasing mutual trust and constructive dissent, c) anticipating potential conflicts with families and working as a team to problem solve them, d) making sure to regularly reevaluate goals as a team, e) working together as a team to break bad news, and f) recognizing the importance of team flexibility.

#### Processes to build a shared mental model

Clinical teams need to have a shared mental model of a patient’s current disease trajectory and the family’s goals in order to develop an appropriate care plan. Like Sara’s neurologists who needed to be updated on the shift in her trajectory, care teams often need to revisit central information to establish a shared mental model. Team leaders can encourage all team members to attend and participate in meetings and rounds, and make sure that team members clearly present key information for each patient [96].

Team leaders can also put processes in place to review relevant changes as a group to ensure that all team members know about changes in patient’s situation. This may mean team members repeat central information multiple times to team members on different shifts through a structured format of communication. Team members who have continuity with patients across multiple attendings, like social workers, can also play an important role in sharing important information about the family with all team members. Team leaders can also reduce the danger of mixed messages by encouraging subspecialists to review treatment recommendations with the attending physician before speaking directly with the family. Pre-meetings before a family meeting provide an excellent opportunity for developing a shared mental model and reviewing the care plan options.

Sharing information helps clinicians make the transition to palliative care goals because clinicians need information in two key areas: how much the patient’s condition has declined over time and whether the family is ready to consider palliative care. Some patients may go through a sudden change (for better or worse) that significantly impacts the appropriateness of cure-seeking treatment and long-term quality of life. In some cases, a clinician may think that a parent is not ready to discuss palliative care based on one conversation, and be unaware that the parent has expressed a change in attitudes to another team member.

#### Mutual trust to encourage constructive dissent

Studies have found that clear shared goals has enhanced effective team work in many settings, including healthcare [86]. Some clinical teams may have a shared goal of providing restorative or cure-directed treatments. However, when these treatments prove to be ineffective, the team may need to reaffirm the goal of providing care consistent with a patient and parent’s goals and values. In addition, team members need to have sufficient trust that each team member is contributing in their own way toward that goal. Groups will tolerate higher levels of conflict and debate if the members feel that they are working together to solve a problem [97].

Clinical teams (and team leaders) should encourage constructive dissent in discussions of treatment options for seriously ill patients encouraging members of the team to speak up about the possibility of initiating palliative care. If the team is biased toward believing that palliative care is only relevant in the very last stage of life, team members can offer the opposing view of what palliative care can offer families earlier in their disease course. Ideally a palliative care representative would attend regular team meetings providing an alternative perspective for what a patient’s care plan could include. Alternatively, teams can have a process where members deliberately provide a devil’s advocate perspective, posing negatively framed question like, “why wouldn’t we consider palliative care for this patient?” to help the team reevaluate whether the current treatment plan is appropriate.

#### Anticipating conflict with families and team problem-solving

Teams should attempt to be aware of conflicts between clinicians and families and develop plans to address the conflict rather than avoiding it [62]. For example, if one team member describes a family as “difficult”, the other team member can ask for more information. In what way are they difficult? What seems to trigger conflicts with this family? What are they most concerned about? What can we do differently to handle this situation? Meetings prior to a family meeting provide opportunities to review previous conflicts, develop consistent team language acknowledging the challenges families have faced, and make explicit the team’s commitment to improving care and communication moving forward [98].

#### Processes for regular reevaluation of goals

For seriously ill patients, clinicians may benefit from periodically stepping back and adopting a deliberative mindset to think about the big picture and reevaluate the pros and cons of the patient’s current treatment goals [18]. What was the patient’s previous baseline? Does the treatment have a meaningful chance of helping the patient? Will the current treatment approach reduce the patient’s quality of life? Are there distressing symptoms that would benefit from palliative care? What are the family members hoping for? Instituting a continuity physician who can pose these questions or establishing a team process to raise these questions for patients who meet a threshold for palliative care may facilitate the consideration of whether subspecialty palliative care is warranted. Having group consensus that this conversation about palliative care should be discussed can be empowering for members of the team lower on the hierarchy who may perceive a value to palliative care consultation, but are reluctant to challenge those in leadership.

#### Sharing serious news as a team

Clinical teams can also work together to establish norms for breaking serious news to families. One study of goals of care discussions with family members of adult patients found that nurses, residents, and physicians thought physicians were the most appropriate team member to initiate discussions of these difficult topics but other team members could also play a role in these discussions [54]. Prior to the family meeting, team members can identify important roles to be fulfilled to ensure that they coordinate with each other during the meeting and do not overlook any important tasks. A “facilitator” for the meeting will keep the meeting on track and consistently check in with the family. An “information giver” will provide relevant medical information, while a “emotional support person” will track the patient and family’s emotion to ensure that is responded to in an empathetic way. Finally, a “recorder” will keep track of relevant information to be shared in the medical record and with the family at the end of the meeting.

#### Importance of team flexibility

Effective teams also need to quickly recognize when a situation has changed, communicate about the change, and adapt their response appropriately [99]. An effective clinical team can be compared to a jazz band. Musicians in a jazz band need to have both the individual ability to play an instrument and the ability and flexibility to respond to what the rest of the band is doing. A jazz musician may be an excellent soloist, but will have trouble playing with a band without these collaboration skills. An individual clinician with excellent communication skills may still confuse or upset a family if the clinician delivers a message that doesn’t fit with what others on the team have said. When team members are aware of each member’s role and responsibilities, they can ensure that all essential tasks are covered and fill in for each other even when an individual team member is overloaded or unavailable.

In some cases, a parent may develop a close rapport with a team member who is not a physician or high in the traditional medical hierarchy. A flexible, collaborative team will be able to follow up on the parent’s concerns even if the trusted team member is not the one who usually initiates palliative care discussions. Collaboration can also play an important role in successful team meetings. One team member may recognize that a parent is confused or overwhelmed and redirect the meeting to address this. A flexible team will follow that cue.

In Fig. 1, on the right hand side, we have distilled previous research on the inhibitors and facilitators of parent/family level regoaling [21–23, 34] with the newly described individual clinician/team level aspects of regoaling represented on the left. By demonstrating the interaction between individual experiences of regoaling for both clinicians and parents with other team members and family members as parallel and conjoined processes, we hope to show the complexity of factors that need to align for a successful involvement of sub-specialty palliative care teams. Individual clinicians’ experience of regoaling is impacted by team-team discussions just as parental regoaling is impacted by discussions with other family members. The articulation of which inhibitor may be causing either the team or family to decline palliative care involvement allows for more targeted strategies to mitigate those inhibitors. Future research can explore the ways that teams and families may respond to facilitators in light of confounding inhibitors for accepting subspecialty palliative care consultation.

## Conclusion

Delayed palliative care referrals for dying children often lead to increased suffering for both the children and their families. Even clinicians who recognize the value that palliative care offers patients and families, and who are frustrated by delays in initiating palliative care, may have trouble initiating the transition. Recognizing potential inhibitors to transitioning to palliative care at the individual and the team level can help clinicians develop individual and team strategies for making the transition to palliative care more effectivity when appropriate. Such strategies can improve the quality of life for these patients and their families.

## Data Availability

Data sharing is not applicable to this article as no datasets were generated or analysed during the current study**.**
